# An update on non-*aureus* staphylococci and mammaliicocci in cow milk: unveiling the presence of *Staphylococcus borealis* and *Staphylococcus rostri* by MALDI-TOF MS

**DOI:** 10.1007/s11259-024-10440-x

**Published:** 2024-06-18

**Authors:** Martina Penati, Fernando Ulloa, Clara Locatelli, Valentina Monistero, Laura Filippone Pavesi, Federica Santandrea, Renata Piccinini, Paolo Moroni, Valerio Bronzo, Maria Filippa Addis

**Affiliations:** 1 Department of Veterinary Medicine and Animal Science - DIVAS, University of Milan, Lodi, Italy; 2 Escuela de Graduados, Facultad de Ciencias Veterinarias, Universidad Austral de Chile, Valdivia, Chile; 3 Laboratorio di Malattie Infettive degli Animali - MiLab, University of Milan, Lodi, Italy

**Keywords:** NASM, *S. borealis*, *S. rostri*, Mastitis, Coagulase-negative staphylococci, Dairy ruminant milk

## Abstract

Non-aureus staphylococci and mammaliicocci (NASM) are the microorganisms most frequently isolated from milk. Given their numerosity and complexity, MALDI-TOF MS is one of the preferred species identification approaches. Nevertheless, reference mass spectra for the novel species *Staphylococcus borealis* were included only recently in the Bruker Biotyper System (MBT) library, and other species of veterinary interest such as *S. rostri* are still absent. This work provides an updated picture of the NASM species found in milk, gained by retrospectively analyzing the data relating to 21,864 milk samples, of which 6,278 from clinical mastitis (CM), 4,039 from subclinical mastitis (SCM), and 11,547 from herd survey (HS), with a spectrum library including both species. As a result, *S. borealis* was the second most frequently isolated NASM (17.07%) after *S. chromogenes* (39.38%) in all sample types, with a slightly higher percentage in CM (21.84%), followed by SCM (17.65%), and HS (14.38%). *S. rostri* was also present in all sample types (3.34%), reaching 8.43% of all NASM in SCM and showing a significant association (*p* < 0.01) with this condition. Based on our findings, the presence of *S. borealis* and *S. rostri* in milk and their potential association with mastitis might have been overlooked, possibly due to the difficulties in differentiating these species from other closely related NASM. Our results indicate that *S. borealis* could be a more frequent contributor to bovine udder infections than previously thought and that *S. rostri* should also not be underestimated considering its significant association with SCM.

**Supplementary Information:**

The online version contains supplementary material available at 10.1007/s11259-024-10440-x.

## Introduction

The bacteria most frequently isolated from cow milk are non-*aureus* staphylococci and mammaliicocci (NASM) (Ruiz-Romero and Vargas-Bello-Pérez 2023; Reydams et al. 2023). However, their specific ability to establish intramammary infection (IMI) and cause mastitis, particularly subclinical mastitis (SCM), is still not entirely clarified. In the last years, the epidemiology of NASM species and their relationships with bovine mammary gland health are getting increasing attention (Lienen et al. 2022; Ruiz-Romero and Vargas-Bello-Pérez 2023). Studies have shown that different NASM behave differently, which can lead to different outcomes in mastitis cases (De Buck et al. 2021; Souza et al. 2023). Therefore, obtaining a precise species identification can be relevant; by clarifying the relationships between species and clinical outcomes, we might be able to ultimately improve animal health (Condas et al. 2017; Ruiz-Romero and Vargas-Bello-Pérez 2023).

The *Staphylococcus* genus encompasses over 72 species (LPSN 2024). Due to limitations in traditional biochemical tests, their laboratory identification can present some challenges. Genotypic methods, such as sequencing of *16s RNA*, *rpoB*, or *hsp60* genes, offer improved accuracy, but are time and labor-demanding, and may fail to differentiate closely related species (Vanderhaeghen et al. 2014; Pain et al. 2020). Being mainly of veterinary interest, the 5 species belonging to the novel genus *Mammaliicoccus* are also neglected in biochemical galleries, and optimized, validated molecular tests are less available. Matrix-assisted laser desorption/ionization time-of-flight mass spectrometry (MALDI-TOF MS) is recognized as a rapid and precise method for the identification of bacteria isolated from milk (Nonnemann et al. 2019). Regular updates of the MALDI-TOF MS library and the inclusion of mass spectra from new species and field isolates can further improve the reliability and accuracy of bacterial identification at the species level, including the NASM group (De Buck et al. 2021). According to the recent literature, the NASM species most commonly isolated from milk are *S. chromogenes*, *S. haemolyticus*, *S. xylosus*, *S. simulans*, and *S. epidermidis* (Vanderhaeghen et al. 2014; De Buck et al. 2021). In line with this, in two recent surveys from our research group the NASM most frequently isolated from clinical mastitis (CM) and SCM quarters were *S. chromogenes* and *S. haemolyticus*. *S. haemolyticus* was also frequently isolated from composite herd survey (HS) milk (Freu et al. 2023; Addis et al. 2024).

Recently, some *S. haemolyticus* were reclassified to the newly defined species *S. borealis*. *S. borealis* was isolated from the skin and blood of six people in Norway and Denmark. These isolates initially appeared to be *S. haemolyticus* due to the extremely high similarity (99.86–99.93%) in the 16 S rRNA gene. However, whole-genome sequencing highlighted the presence of significant differences, with only ~ 88% average nucleotide identity compared to the *S. haemolyticus* type strain. This led to their reclassification as *S. borealis* (Pain et al. 2020). *S. borealis* is both genetically and phenotypically diverse, suggesting that separate bacterial strains live in different host types. Indeed, recent data has indicated that cow strains might be closer to each other than to human strains, suggesting a separate evolution in these hosts (Król et al. 2023). Up to now, *S. borealis* has been isolated from the milk of cows with mastitis in Canada, Poland, and USA (Król et al. 2023; Freu et al. 2023). Thus, understanding the prevalence and distribution of *S. borealis* and its association with mastitis can be of interest for understanding its relationships with mammary gland health.

Although not as frequently as *S. chromogenes* and S. *haemolyticus*, *S. microti* has also been isolated from CM, SCM, and HS milk. Our two surveys indicated percentages around 1% for CM, 4% for SCM and 1.5% for HS milk (Freu et al. 2023; Addis et al. 2024). A recent work reported that the identification by MALDI-TOF MS of NASM isolated from milk as *S. microti* might have been subjected to misclassification (Kløve et al. 2023). By applying whole genome sequencing, these authors analyzed a total of 81 milk isolates identified as *S. microti* by MALDI-TOF MS, finding that these belonged to the *S. rostri* species They revealed a strong genetic intra-herd conservation, implying the bovine adaptation of *S. rostri* and its ability to specifically spread within this host. Accordingly, the existence of *S. rostri* and its relevance for bovine mammary gland health might have been overlooked so far.

Based on these premises, this work aims to provide an updated picture of the NASM species distribution by retrospectively analyzing the results obtained by MALDI-TOF MS on CM, SCM, and HS milk, throughout one year by MALDI-TOF MS against a library including spectra for *S. borealis* and *S. rostri*, to shed light on their presence in dairy cow milk. The study was carried out in northern Italy, an area characterized by a high dairy herd density playing a relevant role in the European dairy production (CLAL 2024).

## Materials and methods

### Milk samples and contributing herds

We retrieved the bacteriological results related to the milk samples sent to the Animal Infectious Disease Laboratory (MiLab), University of Milan, between December 2022 and November 2023. The study included 106 dairy farms from Northern Italy housing Holstein-Friesian cows in free-stall barns, which sent CM, SCM and HS milk samples. For CM, trained personnel, guided by established clinical criteria (Adkins et al. 2017), identified affected mammary quarters through visual examination of first milk streams for abnormal characteristics such as flakes, clots, discolouration, or watery consistency, alongside swelling, redness, and pain in the udder. The SCM originated from cows with elevated SCC in the latest DHI recording, which underwent further evaluation with the California Mastitis Test (CMT). Samples from quarters testing positive for CMT without clinical signs were classified SCM according to established criteria (Adkins et al. 2017). Finally, composite HS samples were collected during total herd samplings to assess herd-level prevalence of infectious pathogens. All personnel adhered to National Mastitis Council (NMC) protocols (Adkins et al. 2017) during sample collection, employing pre-dipping disinfectant and alcohol-containing wipes for teat disinfection before taking the samples, and promptly sending them to the laboratory in a refrigerated box.

### Bacteriological analysis of milk

Milk samples were cultured following the NMC (Middleton et al. 2017). Briefly, 10 µL of milk was spread on blood agar (Microbiol, Cagliari, Italy) and incubated aerobically at 37 °C for 24–48 h. Plates were then interpreted and classified as positive, contaminated, or negative according to NMC criteria (Adkins et al. 2017). From the positive plates, the isolated colonies were identified using MALDI-TOF MS (Rosa et al. 2022) and the spectra were processed with the MALDI Biotyper (MBT) Compass® Library Revision H (2022) (Bruker Daltonik GmbH, Bremen, Germany). A custom MALDI-TOF MS library was used for this analysis. It included two strains of *S. rostri* that were previously identified by sequencing the *rpoB* gene (Locatelli et al. 2013).

### Data analysis

Since November 30, 2022, when the MBT library was updated with the mass spectra for *S. borealis* and *S. rostri*, 21,864 milk samples were analyzed, including 6,278 CM, 4,039 SCM, and 11,547 composite HS samples. Information on farm ID, sampling dates, sample types, microbiological results, and identification scores were stored in a Microsoft Access database, together with information on clinical/subclinical status. The data used for this work was extracted using Microsoft Excel (Microsoft Office, version 16.82, 2024). Pivot tables and built-in Excel functions were employed to generate descriptive statistics. Statistical analysis was carried out using SPSS 28.0 (IBM, SPSS, Armonk, USA). A multinomial logistic regression model was used to compare the relative prevalence of NASM species in CM and SCM. The subclinical outcome served as the reference category for estimation of parameters using Wald statistics. Significance for the analyses was determined with p-values < 0.05 and < 0.01.For estimating the within-farm distribution of NASM species, only farms sending at least 10 samples of which one positive for NASM were considered.

## Results

### Microbiology results according to the sample type: quarter and composite milk samples

The microbiological culture results obtained on the 21,864 milk samples are summarized in Table 1 according to the sample type. Out of 6,278 CM milk samples collected from 96 herds, 63.57% (3,991) were positive, of which 17.66% (705) were positive for NASM. Out of 4,039 SCM milk samples collected from 34 herds, 69.03% (2,788) of samples were positive, of which 31.49% (878) for NASM. Out of 11,547 composite HS milk samples collected from 33 herds, 32.37% (3,738) were positive, of which 38.5% (1,439) for NASM (Table 1).

**Table 1:** Bacteriological culture results obtained for all the milk samples considered in this study, according to their respective categories

Results	CM quarter milk	SCM quarter milk	HS composite milk	Total
Negative	1,578 (25.14%)	892 (22.08%)	7,113 (61.6%)	9,583 (43.83%)
Contaminated	709 (11.29%)	359 (8.89%)	696 (6.03%)	1,764 (8.07%)
Positive	3,991 (63.57%)	2,788 (69.03%)	3,738 (32.37%)	10,517 (48.10%)
Of which NASM^a^	705 (17.66%)	878 (31.49%)	1,439 (38.5%)	3,022 (28.73%)
Total	6,278	4,039	11,547	21,864

*CM* clinical mastitis, *SCM* subclinical mastitis, *HS* herd survey

^a^NASM percentage on the total positive samples in each category

### NASM species isolated from mastitis and herd survey milk and identified by MALDI-TOF MS with the MBT System

Species information could be retrieved for 3,022 NASM isolates. A total of 19 distinct NASM species were identified and are reported in Table 2 with their respective average MALDI-TOF MS Log score. *S. chromogenes* was the first species with 39.38%, followed by *S. borealis* (17.07%) and *M. sciuri* (9.89%). The average identification Log score was 2.08, with a minimum of 1.77 for *S. gallinarum* and a maximum of 2.43 for *S. rostri*. Across all log score cutoffs tested (1.7, 2.0, and even a stricter 2.3), *S. chromogenes* and *S. borealis* were the first and second most prevalent NASM.

**Table 2:** Total number of isolates for each NASM species with their respective average MALDI-TOF MS Log score

NASM species	Number of isolates (%)	Average Log score
*S. chromogenes*	1,190 (39.38%)	2.14
*S. borealis*	516 (17.07%)	2.13
*M. sciuri*	299 (9.89%)	1.95
*S. xylosus*	262 (8.67%)	1.98
*S. epidermidis*	224 (7.41%)	2.05
*S. haemolyticus*	197 (6.52%)	1.93
*S. rostri*	101 (3.34%)	2.43
*S. simulans*	64 (2.12%)	2.07
*S. arlettae*	52 (1.72%)	1.82
*S. equorum*	46 (1.52%)	2.02
*S. saprophyticus*	19 (0.63%)	2.00
*S. agnetis/hyicus*^a^	16 (0.53%)	2.11
*S. gallinarum*	14 (0.46%)	1.77
*S. capitis*	8 (0.26%)	1.93
*S. cohnii*	6 (0.2%)	1.93
*S. hominis*	3 (0.1%)	1.97
*S. succinus*	3 (0.1%)	1.88
*S. microti*	1 (0.03%)	2.29
*S. schleiferi*	1 (0.03%)	2.28
**Total**	**3,022 (100.00%)**	**2.08**

^a^MALDI-TOF MS does not differentiate these two species

Table 3 reports the NASM species identified according to the sample type. The three most frequent NASM in CM quarters were *S. chromogenes* (24.82%), *S. borealis* (21.84%), and *M. sciuri* (20.57%), with similar prevalence levels. In SCM quarters, *S. chromogenes* was also the most prevalent species (31.78%) followed by *S. borealis*, albeit with slightly lower percentages (17.65%). *S. xylosus* was third (10.59%). In composite milk, *S. chromogenes* was largely predominant, representing over half (51.15%) of the isolates, once again followed by *S. borealis* (14.38%). Among other NASM species, *M. sciuri*, *S. equorum*, and *S. arlettae* were significantly more present in CM, while *S. xylosus*, *S. epidermidis*, and *S. rostri* were significantly more present in SCM samples (*p* < 0.01; Supplementary Table).

**Table 3:** Distribution of NASM species across sample categories

CM quarter milk	*N* (%)	SCM quarter milk	*N* (%)	HS composite milk	*N* (%)
*S. chromogenes*	175 (24.82%)	*S. chromogenes*	279 (31.78%)	*S. chromogenes*	736 (51.15%)
*S. borealis*	154 (21.84%)	*S. borealis*	155 (17.65%)	*S. borealis*	207 (14.38%)
*M. sciuri*	145 (20.57%)	*S. xylosus*	93 (10.59%)	*S. epidermidis*	114 (7.92%)
*S. xylosus*	73 (10.36%)	*S. epidermidis*	83 (9.45%)	*S. xylosus*	96 (6.67%)
*S. haemolyticus*	54 (7.66%)	*S. haemolyticus*	74 (8.43%)	*M. sciuri*	93 (6.46%)
*S. epidermidis*	27 (3.83%)	*S. rostri*	74 (8.43%)	*S. haemolyticus*	69 (4.79%)
*S. equorum*	23 (3.26%)	*M. sciuri*	61 (6.95%)	*S. simulans*	39 (2.71%)
*S. arlettae*	19 (2.7%)	*S. simulans*	16 (1.82%)	*S. arlettae*	24 (1.67%)
*S. simulans*	9 (1.28%)	*S. equorum*	12 (1.37%)	*S. rostri*	21 (1.46%)
*S. gallinarum*	6 (0.85%)	*S. arlettae*	9 (1.03%)	*S. equorum*	11 (0.76%)
*S. rostri*	6 (0.85%)	*S. saprophyticus*	6 (0.68%)	*S. agnetis/S. hyicus*^a^	8 (0.56%)
*S. saprophyticus*	6 (0.85%)	*S. gallinarum*	5 (0.57%)	*S. saprophyticus*	7 (0.49%)
*S. agnetis/S. hyicus*^a^	5 (0.71%)	*S. capitis*	3 (0.34%)	*S. cohnii*	5 (0.35%)
*S. succinus*	2 (0.28%)	*S. agnetis/S. hyicus*^a^	3 (0.34%)	*S. capitis*	4 (0.28%)
*S. capitis*	1 (0.14%)	*S. cohnii*	1 (0.11%)	*S. gallinarum*	3 (0.21%)
*S. cohnii*	-	*S. hominis*	1 (0.11%)	*S. hominis*	2 (0.14%)
*S. hominis*	-	*S. microti*	1 (0.11%)	*S. microti*	-
*S. microti*	-	*S. schleiferi*	1 (0.11%)	*S. schleiferi*	-
*S. schleiferi*	-	*S. succinus*	1 (0.11%)	*S. succinus*	-
**Total**	**705**	**Total**	**878**	**Total**	**1,439**

*CM* clinical mastitis, *SCM* subclinical mastitis, *HS* herd survey

^a^MALDI-TOF MS does not differentiate these two species

### NASM distribution in farms

**Fig. 1:**
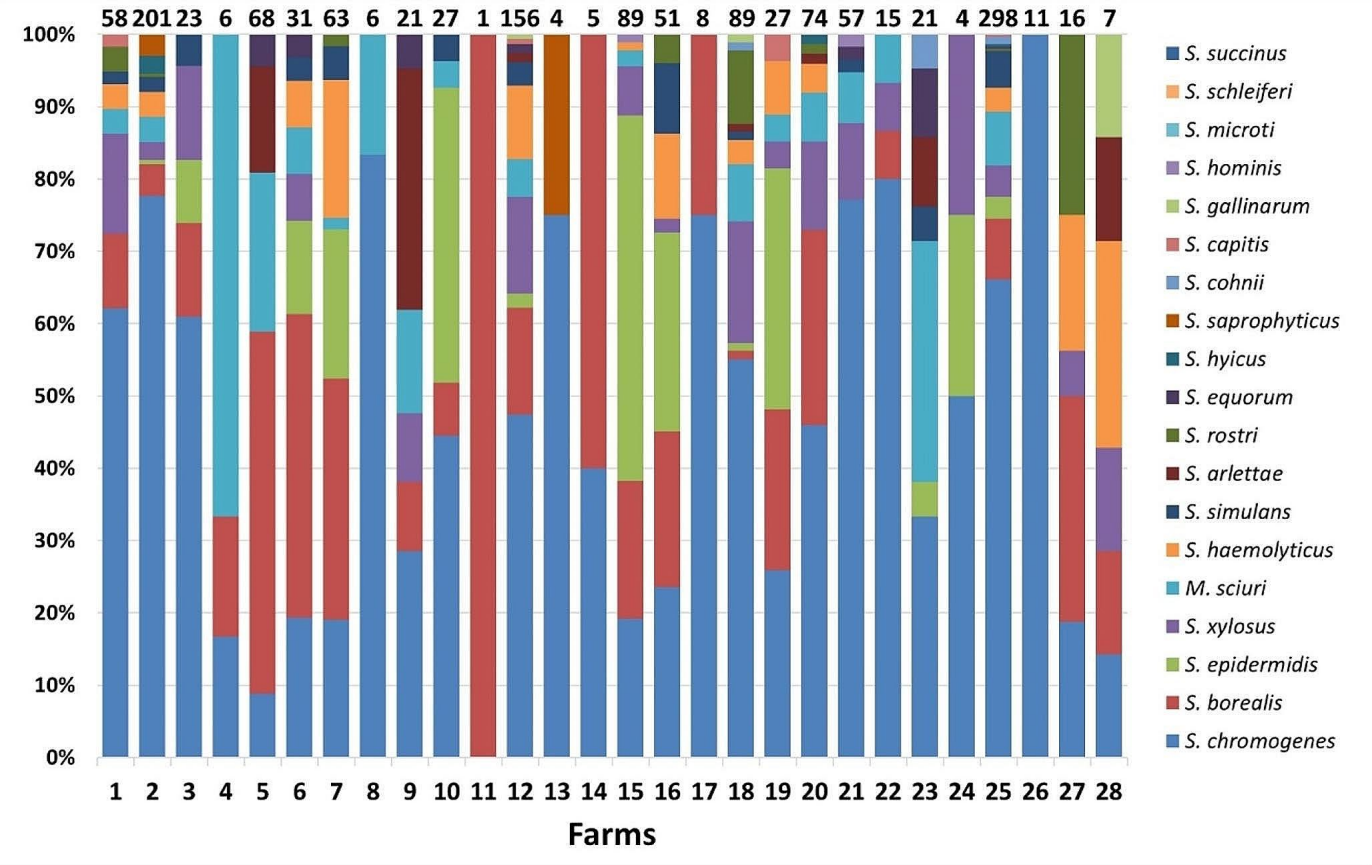
Relative percent distribution of NASM species identified in composite HS milk according to the contributing farm. Only positive farms sending at least 10 milk samples are reported. The numbers at the top of the figure indicate the number of isolates per farm

A varied distribution of NASM species was observed in the farms sending composite HS samples (Fig. 1). *S. chromogenes* was isolated in all but one farm (96.43%) and was the main NASM in over half of the farms (57.14%). Notably, *S. borealis* was the second species, being present in 22 of 28 farms (78.57%) and representing the main NASM in 6 of 28 farms (21.42%). Other frequently identified NASM were *M. sciuri* (64,29%), *S. xylosus* (60,71%), *S. epidermidis* (46,73%), *S. haemolyticus* (46,43%) and *S. simulans* (42,86%). *S. rostri* was isolated in 8 out of 28 farms (28.57%) but it was never the most prevalent NASM species found in the herd.

## Discussion

The relevance of NASM for animal welfare and milk quality is increasingly recognized in light of their association with CM and SCM in modern dairy farms (Schukken et al. 2009; De Buck et al. 2021; Ruiz-Romero and Vargas-Bello-Pérez 2023). This retrospective study investigated the prevalence of various NASM species identified by the MBT System with the aim of gathering novel information on *S. borealis* and *S. rostri*.

In the course of one year, we identified 19 different NASM species on a total of 21,864 samples including CM, SCM and HS milk. Of these, *S. borealis* was the second most common species after *S. chromogenes* across sample types. Therefore, our results indicate that *S. borealis* might be a more frequent contributor to bovine udder infections than previously thought, confirming the recent findings from our group in the US (Freu et al. 2023) and in line with the increasingly reported presence of *S. borealis* within cattle population as a possible cause of IMI (Król et al. 2023).

In our previous retrospective studies (Freu et al. 2023; Addis et al. 2024), S. *haemolyticus* was the second most prevalent species after *S. chromogenes*. A recent systematic review by Ruiz-Romero and Vargas-Bello-Pérez (2023) also reported *S. haemolyticus* as the second most frequently isolated NASM. However, the identification of *S. haemolyticus* has created significant challenges in the past due to the elevated genomic variability combined with a high similarity of its marker genes with other species (Wanecka et al. 2018; Rosa et al. 2022). Recently, whole genome sequencing of *S. haemolyticus* has uncovered a significant phylogenetic distance among isolates, leading to the first description of *S. borealis* as a distinct species (Pain et al. 2020). Accordingly, isolates initially identified as *S. haemolyticus* were later confirmed to be *S. borealis* (Król et al. 2023). Therefore, the high similarity of its marker genes to *S. haemolyticus* and the absence of reference spectra in the MBT database for *S. borealis* have likely led to its previous misclassification as *S. haemolyticus*, underestimating the presence of *S. borealis* and overestimating *S. haemolyticus* (Freu et al. 2023). The present work provides further indications that *S. borealis* could indeed be a common cause of bovine mastitis, in line with our findings in the US for CM (Freu et al. 2023), thus underscoring the importance of distinguishing it from *S. haemolyticus* for an accurate understanding of its role in CM and SCM. Its monitoring and characterization can also be important for the potential ability to spread antimicrobial resistance genes, as multidrug-resistant *S. borealis* strains have been already detected in pigs (Abdullahi et al. 2023). In the future, it will be important to carry out confirmatory sequencing of bovine *S. borealis* and *S. haemolyticus* isolates to strengthen this conclusion. As long as the discriminatory power of MALDI-TOF MS for the two species *S. borealis* and *S. haemolyticus* is not firmly established, some caution will be necessary in the interpretation of results, especially in light of the low average identification Log scores still being observed for *S. haemolyticus*.

Another interesting finding was the emergence of *S. rostri*, and of its significant association with SCM, after updating our library with in-house spectra for his species. Different studies suggested that creating an internal mass spectrum library of accurately identified reference strains can significantly improve the reliability of isolate identification by MALDI-TOF MS, especially when commercially available databases include a limited number of reference spectra or when some species are absent (Cassagne et al. 2011; Posteraro et al. 2012; Kolecka et al. 2014). In the past, *S. rostri* has been identified in various hosts, including pigs (Vanderhaeghen et al. 2012) and water buffaloes (Locatelli et al. 2013). In cows, previous evidence suggested that *S. rostri* may serve as a pathogen contributing to mastitis and that it can persist on farms through cow-to-cow transmission or within environmental reservoirs (Jenkins et al. 2019; Kløve et al. 2023). As in the case of *S. borealis*, differentiating *S. rostri* from *S. microti* and *S. muscae* is hampered by genetic similarity as well as by limitations in existing databases (Rosa et al. 2022; Kløve et al. 2023). In our previous retrospective studies, *S. microti* was the sixth most prevalent NASM in SCM and the ninth and tenth, respectively, in CM cases from US and Italy (Freu et al. 2023; Addis et al. 2024). In the present study, *S. rostri* accounted for 8.43% of all NASM identified in SCM, while *S. microti* was isolated only once, suggesting that previously reported *S. microti* might have actually been *S. rostri* and that the reported prevalence data for *S. microti* might have been overestimated. Previous studies identified antibiotic resistance in *S. rostri* (Wuytack et al. 2019), and monitoring resistance patterns in isolates from bovine mastitis could also be an area for future research.

Regarding the potential association of NASM with mastitis cases, *S. borealis* was detected more frequently in CM, but it was not preferentially associated with SCM or CM. In HS, *S. borealis* emerged as the second most prevalent species after *S. chromogenes*, with a widespread presence on most farms. This finding suggests a similar role in udder health compared to other NASM. On the other hand, *S. rostri* was significantly associated with SCM. In HS, *S. rostri* displayed a lower prevalence compared to the previous two NASM.

In conclusion, the prevalence of *S. borealis* and *S. rostri* has likely been underestimated due to a lack of capacity to differentiate them from closely related bacteria. The MBT database, initially focused on human pathogens (Tomazi et al. 2014), is gradually expanding its coverage of veterinary isolates. The continued development of this resource and, possibly, its integration with characterized bovine isolates, will be crucial for accurate identification of NASM species. By enabling a more precise exploration of the specific impacts of different NASM species on udder health, future research can inform more targeted farm management strategies to enhance animal health and milk quality.

### Electronic supplementary material

Below is the link to the electronic supplementary material.

**Supplementary Table** Results of the application of a multinomial logistic regression model, using as a reference category the subclinical outcome, estimating the parameters with Wald statistics, for assessing the relationships with the NASM species.

## Data Availability

No datasets were generated or analysed during the current study.
