# Supplementary material for: An update on non-aureus staphylococci and mammaliicocci in cow milk: unveiling the presence of Staphylococcus borealis and Staphylococcus rostri by MALDI-TOF MS
Source: Vet Res Commun. 2024 Jun 18;48(4):2555–61. doi: 10.1007/s11259-024-10440-x (PMC11315763; doi:10.1007/s11259-024-10440-x)
Supplement: Supplementary file 1 — Supplementary Table Results of the application of a multinomial logistic regression model, using as a reference category the subclinical outcome, estimating the parameters with Wald statistics, for assessing the relationships with the NASM species. [file 11259_2024_10440_MOESM1_ESM.docx]

**An update on non-aureus staphylococci and mammaliicocci in cow milk: unveiling the presence of *Staphylococcus borealis* and *Staphylococcus rostri* by MALDI-TOF MS**

Martina Penati^1^, Fernando Ulloa^2^, Clara Locatelli^1^, Valentina Monistero^1^, Laura Filippone Pavesi^1^, Renata Piccinini^1,3^, Paolo Moroni^1,3^, Valerio Bronzo^1,3^, Maria Filippa Addis^1,3^*

^1^Department of Veterinary Medicine and Animal Science - DIVAS, University of Milan, Lodi, Italy

^2^Escuela de Graduados, Facultad de Ciencias Veterinarias, Universidad Austral de Chile, Valdivia, Chile

^3^Laboratorio di Malattie Infettive degli Animali - MiLab, University of Milan, Lodi, Italy

*Corresponding author. E-mail address: filippa.addis@unimi.it (M. F. Addis)

**Supplementary Table** Results of the application of a multinomial logistic regression model, using as a reference category the subclinical outcome,

estimating the parameters with Wald statistics, for assessing the relationships with the NASM species.

| Mast_cod | | b | Std. Err. | Wald | Ggl | *P* value | Exp(b) | 95% conf. interval for Exp(b) | |
| --- | --- | --- | --- | --- | --- | --- | --- | --- | --- |
|  |  |  |  |  |  |  |  | Lower limit | Upper limit |
| CM | Intercept | -0.242 | 0.156 | 2.398 | 1 | 0.121 |  |  |  |
|  | [NASM = *S. arlettae*] | 0.989 | 0.434 | 5.201 | 1 | **0.023** | 2.689 | 1.149 | 6.294 |
|  | [NASM = *S. borealis*] | 0.236 | 0.193 | 1.485 | 1 | 0.223 | 1.266 | 0.866 | 1.849 |
|  | [NASM = *S. capitis*] | -0.856 | 1.165 | 0.540 | 1 | 0.462 | 0.425 | 0.043 | 4.168 |
|  | [NASM = *S. chromogenes*] | -0.224 | 0.184 | 1.490 | 1 | 0.222 | 0.799 | 0.557 | 1.145 |
|  | [NASM = *S. epidermidis*] | -0.881 | 0.271 | 10.551 | 1 | **0.001** | 0.414 | 0.244 | 0.705 |
|  | [NASM = *S. equorum*] | 0.893 | 0.389 | 5.269 | 1 | **0.022** | 2.442 | 1.139 | 5.233 |
|  | [NASM = *S. gallinarum*] | 0.424 | 0.625 | 0.461 | 1 | 0.497 | 1.529 | 0.449 | 5.208 |
|  | [NASM = *S. haemolyticus*] | -0.073 | 0.238 | 0.094 | 1 | 0.759 | 0.930 | 0.583 | 1.481 |
|  | [NASM = *S. hyicus*] | 0.753 | 0.747 | 1.016 | 1 | 0.313 | 2.123 | 0.491 | 9.178 |
|  | [NASM = *S. rostri*] | -2.270 | 0.452 | 25.185 | 1 | **0.000** | 0.103 | 0.043 | 0.251 |
|  | [NASM = *S. saprophyticus*] | 0.242 | 0.598 | 0.164 | 1 | 0.686 | 1.274 | 0.394 | 4.114 |
|  | [NASM = *S. sciuri*] | 1.108 | 0.218 | 25.715 | 1 | **0.000** | 3.028 | 1.973 | 4.647 |
|  | [NASM = *S. simulans*] | -0.333 | 0.445 | 0.561 | 1 | 0.454 | 0.717 | 0.300 | 1.714 |
|  | [NASM = *S. succinus*] | 0.935 | 1.235 | 0.574 | 1 | 0.449 | 2.548 | 0.227 | 28.652 |
|  | [NASM = *S. xylosus*] | -0.989 | 0.434 | 5.201 | 1 | **0.023** | 0.372 | 0.159 | 0.870 |

*S. cohonii, S. hominis, S. microti, S. schleiferi* were excluded from the table as these species were isolated only once.
